# Thermodynamic database for the Co-Pr system

**DOI:** 10.1016/j.dib.2015.12.035

**Published:** 2016-01-02

**Authors:** S.H. Zhou, M.J. Kramer, F.Q. Meng, R.W. McCallum, R.T. Ott

**Affiliations:** a The Critical Materials Institute, Ames Laboratory, DOE, USA; b Division of Materials Sciences and Engineering, Ames Laboratory, USDOE, USA; c Department of Materials Science and Engineering, Iowa State University, USA

**Keywords:** Thermodynamic database of Co-Pr, Solution calorimeter measurement, Phase diagram Co-Pr

## Abstract

In this article, we describe data on (1) compositions for both as-cast and heat treated specimens were summarized in Table 1; (2) the determined enthalpy of mixing of liquid phase is listed in Table 2; (3) thermodynamic database of the Co-Pr system in TDB format for the research articled entitle Chemical partitioning for the Co-Pr system: First-principles, experiments and energetic calculations to investigate the hard magnetic phase W.

Specifications table

**Table:** 

Subject area	Materials science
More specific subject area	Thermodynamics
Type of data	Table, Thermo-calc format TDB file
How data was acquired	Electron-probe microanalysis (EPMA), solution calorimeter
Data format	Raw,
Experimental factors	Samples were arc-melted 5 times and heat treated at different conditions.
Experimental features	EPMA: identify phases. Solution calorimeter: measure the heat effect.
Data source location	Division of Materials Sciences and Engineering, Ames Laboratory
Data accessibility	Data is with this article

Value of the data

• The phase diagram data is essential for determining the thermodynamic database.
• The experimental enthalpy of mixing of the liquid is one of keys for computing the Gibbs free energy of the liquid phase.
• The thermodynamic database evaluating on basis of phase diagram data, thermochemical data and first-principle data can be used to calculate thermodynamic properties, phase diagram and nonequilibrium chemical partitioning phase transformation.

## 1 Data

Table 1 gives the observed phases for both as-cast and heat treated alloys. Table 2 lists the enthalpy of mixing of Co-Pr liquid. The Supplementary file is the thermodynamic database of the binary Co-Pr system.

## 2 Experimental design, materials and methods

### 2.1 Experimental determined phase compositions and enthalpy of mixing of liquid

Alloy test specimens of Co-*x* at% Pr (*x*=10.5–54.0) were prepared by arc melting pure elements (99.9 Co and 99.99 Pr, wt% [1]) on a copper hearth in an argon atmosphere. Each alloy specimen was arc-melted and flipped 5 times to ensure homogeneity, and then drop cast into a 6 mm diameter Cu mold. The as-cast samples were wrapped in tantalum foil and treated at temperature(K)+time(hours) as listed in Table 1 in an argon atmosphere and quenched into water. Both as-cast and heat treated specimens were characterized using EPMA with wavelength dispersive spectroscopy, as summarized in Table 1.

The enthalpy of mixing of Co-Pr liquid was measured with solution calorimeter using the isoperibolic procedure. Details concerning the calorimeter setup and the measurement procedure have been described previously [2]. The measurements were carried out under argon gas at atmospheric pressure and constant temperature at 1327 K with continuous stirring. Δ*H*(*x*_Co_) was measured by successive additions of Co component from room temperature to a liquid Pr bath contained in a Ta crucible. The temperature change was measured by a K-type thermopile situated directly below the crucible. The enthalpy of solution corresponds to the area under the Δ*T* vs. time curve. For each series of measurements, the calorimeter was calibrated by dropping pure Pr samples into the bath and their heat content was taken from database by Dinsdale [3]. After measurement, the sample was easily separated from crucible indicating no obvious reaction between sample and crucible. As listed in Table 2, the measurements for the enthalpy of mixing of liquid were performed for composition up to *x*_Co_=0.486. The standard deviation of the calibrations was of the order of ±3%.

### 2.2 Thermodynamic database

Thermodynamic database was assessed with all relevant experimental and first-principles calculations. The database in TDB format can be used to thermodynamic properties, phase diagram and Chemical Partitioning-Temperature-Phase Transformation calculation [4].

## Figures and Tables

**Table 1:** EPMA measured phase compositions.

Nominal Alloy x_Pr_	Treated at T(K)+hours	x_Pr_							
		Co-hcp	Co_17_Pr_2_-α	Co_5_Pr-D2_d_	Co_19_Pr_5_-β	Co_7_Pr_2_-χ	Co_3_Pr-δ	Co_2_Pr-C15	Co_17_Pr_20_-ϕ

0.105	as-cast	0.0058	0.108	0.143	–	–	–	–	–
0.167	as-cast	–	–	0.158	–	0.218	–	–	–
1194+168	–	–	0.164	–	–	–	0.322	–
0.208	as-cast	–	–	0.163	0.207	0.220	0.245	0.326	–
773+336	–	–	0.165	–	0.220	0.245	–	–
0.222	as-cast	–	–	0.158	0.209	0.216	0.240	0.305	–
1173+168	–	–	–	–	0.222	0.250	–	–
1000+168	–	–	0.165	–	0.220	0.235	–	–
0.54	as-cast	–	–	–	–	–	–	0.324	0.523
834+396	–	–	–	–	–	–	0.332	0.538

**Table 2:** Experimental values of the enthalpy change during dissolution of solid Co and Pr at 298 K in liquid Pr at 1327 K and calculated enthalpy of mixing to reference states: Co-liq and Pr-liq.

Added n_Pr_	Added n_Co_	Comp.	ΣδΔ*H*	Δ*H*
In mole		x_Co_	J/mol	

0.10701	0	0	0	0
0.00558	0.01201	0.096	−5697.59	−5697.59
0.00613	0.01708	0.197	−5314.57	−11012.2
0	0.01738	0.281	−3049.74	−14061.9
0	0.01515	0.342	−1383.32	−15445.2
0	0.01857	0.403	−1153.14	−16598.4
0	0.01903	0.455	−698.562	−17296.9
0	0.01309	0.486	−412.618	−17309.5
